# PD-1 abrogates the prolonged persistence of CD8^+^ CAR-T cells with 4-1BB co-stimulation

**DOI:** 10.1038/s41392-020-00277-6

**Published:** 2020-08-25

**Authors:** Feng Li, Zhen Zhang, Yujing Xuan, Daiqun Zhang, Jinyan Liu, Aitian Li, Shumin Wang, Ting Li, Xiaojuan Shi, Yi Zhang

**Affiliations:** 1 Biotherapy Center, The First Affiliated Hospital of Zhengzhou University, Zhengzhou, Henan China; 2 Cancer Center, The First Affiliated Hospital of Zhengzhou University, Zhengzhou, Henan China; 3 State Key Laboratory of Esophageal Cancer Prevention & Treatment, Zhengzhou, Henan China; 4 Henan Key Laboratory for Tumor Immunology and Biotherapy, Zhengzhou, Henan China; 5 School of Life Sciences, Zhengzhou University, Zhengzhou, Henan China

**Keywords:** Tumour immunology, Immunotherapy

**Dear Editor,**

The central memory differentiation critically dictates CAR-T cell persistence, which is closely associated with the therapeutic effectiveness in the clinic. It is well accepted that 4-1BB co-stimulation is superior over CD28 to promote the central memory differentiation and persistence of CAR-T cells.^1^ However, it is also noticed that CAR-T cells with either co-stimulations are comparably differentiated and persisted,^2^ especially under immunosuppressive conditions. Factors contributing to the discrepancy remain unknown. Mounting evidences show that programmed cell death protein 1 (PD-1) directly affects memory differentiation of T cells upon PD-L1 engagement,^3^ in addition to limiting proliferation. It is unclear whether PD-1 is involved in the diminished difference in memory differentiation and persistence between CAR-T cells with different co-stimulations. Herein, we examined the memory differentiation of CAR-T cells when PD-1 is activated or not, and consequently the persistence and anti-tumor effects.

CD8^+^ T cells were used in this study because of their direct cytotoxicity against malignant cells. As shown in Fig. 1a and Supplementary Fig. 1a, CD8^+^ CAR-T cells incorporated with CD28 (28ζ) or 4-1BB (BBζ) co-stimulations were generated using a human-sourced antibody specific for mesothelin. CAR-T cells demonstrated potent antigen-dependent cytotoxicity (Fig. 1b, c and Supplementary Fig. 1b). Compared with 28ζ cells, BBζ cells better preserved the central memory subsets (CD45RA^−^CD62L^+^) and showed improved persistence during in vitro expansion (*P* < 0.05; Supplementary Fig. 1c, d). These results suggest that the basal activity of 4-1BB co-stimulation promotes central memory differentiation and persistence of CAR-T cells.

**Fig. 1:**
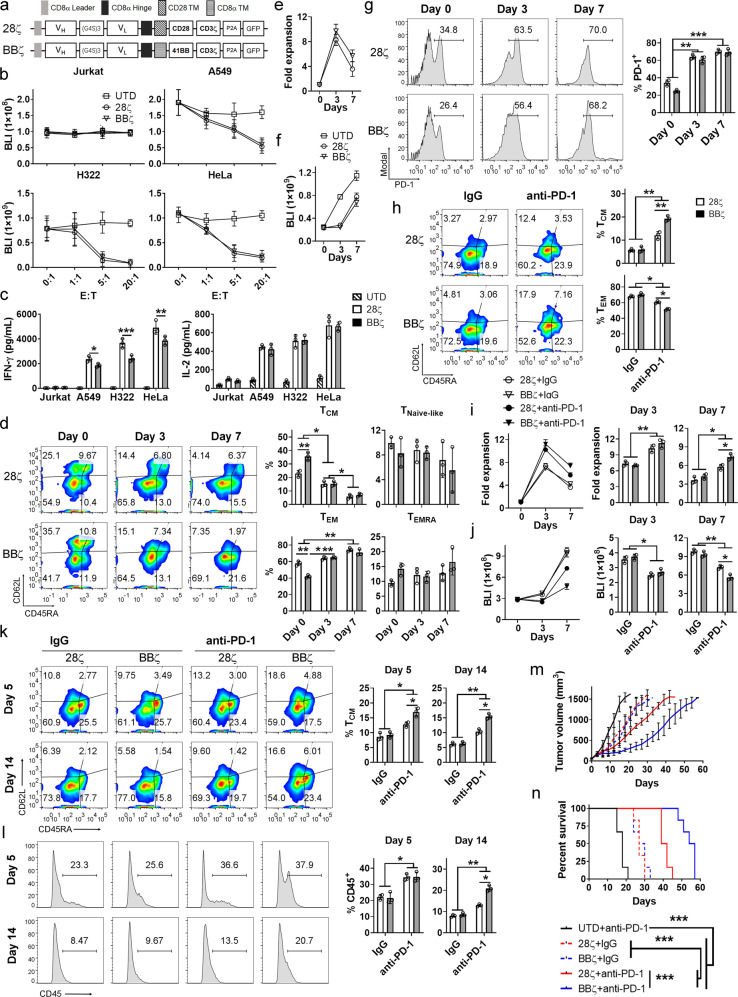
PD-1 diminishes the advantages in central memory accumulation and long-term anti-tumor effects of CD8^+^ CAR-T cells with 4-1BB co-stimulation. **a**–**c** Construction and characterization of CD8^+^ CAR-T cells. Structures of 28ζ and BBζ CAR (**a**). CD8^+^ untransduced (UTD), 28ζ and BBζ Τ cells were incubated with target cells at the indicated E:T ratios for 24 h, after which target cell viability was determined according to bioluminescence (BLI) intensity (**b**). Tumor cells were incubated with listed T cells at E:T = 1:1 for 24 h, after which the supernatants were collected, and ELISA was performed to determine IFN-γ and IL-2 secretion (**c**). **d**–**g** Activation-induced memory differentiation and PD-1 expression of CD8^+^ CAR-T cells. HeLa cells expressing luciferase were adhered to the wells overnight, then purified CD8^+^ CAR-T cells were added at E:T = 1:10 without exogenous cytokines. The differentiation statuses of CAR-T cells were determined and the ratios of naive (T_Naive-like_; CD45RA^+^CD62L^+^), central memory (T_CM_; CD45RA^−^CD62L^+^), effector memory (T_EM_; CD45RA^−^CD62L^−^), and most differentiated T (T_EMRA_; CD45RA^+^CD62L^−^) cells were compared on day 0, 3, and 7 post-co-incubation (**d**). CAR-T cell expansions were determined on days 0, 3, and 7. At the indicated time point, suspended cells were collected, and dead cells were excluded by Trypan Blue staining. Live cells were then counted and compared with those of 28ζ cells on day 0 (**e**). The long-term tumoricidal activity of CAR-T cells was monitored via BLI assays (**f**). On days 0, 3, and 7, CAR-T cells were collected and PD-1 expression was determined using FACS (**g**). **h**–**j** PD-1 blockade increased T_cm_ subset and the persistence of CD8^+^ CAR-T cells. Luciferase-expressing HeLa cells were adhered to the well surface overnight, after which purified CD8^+^ CAR-T cells were added at E:T = 1:10 without exogenous cytokines. IgG and anti-PD-1 antibody were repeatedly added on days 0, 3, and 6 at a concentration of 20 μg/mL. On day 7 post-incubation, CAR-T cells were collected and their differentiation statuses were determined (**h**). CAR-T cell proliferation was determined on days 0, 3, and 7 and compared (**i**). Tumor cell proliferation was monitored via BLI assay and statistically analyzed (**j**). **k**, **l** PD-1 blockade enhances the central memory differentiation of CD8^+^ CAR-T cells in vivo. HeLa cells were subcutaneously inoculated into SCID-Beige mice. Ten days later, 5 × 10^6^ CD8^+^ CAR-T cells were injected once through the tail vein and the indicated antibodies were injected intraperitoneally four times every 3 days (*n* = 3 per group). On days 5 and 14 post-CAR-T cell infusion, tumor tissues were isolated, and single-cell suspensions were prepared. After excluding dead cells using Fixable Viability Dye eFluor™ 660, the differentiation status (**k**) and intratumoral accumulation (**l**) of CAR-T cells were determined between mice that received non-specific IgG or anti-PD-1 neutralizing antibody. **m**, **n** PD-1 blockade improved the anti-tumor effects of CD8^+^ CAR-T cells in vivo. Mice (*n* = 6 in each group) with established xenograft tumors were injected with 5 × 10^6^ CD8^+^ UTD or CAR-T cells once on day 0 and six times with IgG or anti-PD-1 antibody every 3 days after T cell infusion. Tumor volumes were calculated every 3 days after T cell injection (**m**). And the survival curves for mice with different treatments were demonstrated (**n**). Data presented as the means ± SD are representative of three independent experiments on CAR-T cells collected from at least three healthy donors. Statistical analysis was performed using *t*-test or ANOVA. Log-rank test was used to compare the difference of survival among mice. *P*-values < 0.05 are considered statistically significant. *P < 0.05; **P < 0.01; ***P < 0.005

Next, we purified CAR-T cells (Supplementary Fig. 2) and analyzed their persistence and differentiation upon engagement with target cells. CAR-T cells with different co-stimulations were comparably activated (Supplementary Fig. 3a, b). Consistent, a study on mesothelin-specific CAR-T cells noted that co-stimulations had little impact on the direct cytotoxicity of engineered T cells.^4^ Compared with 28ζ counterparts, the activated BBζ cells did not show superior central memory enrichment, persistence, and tumoricidal activity neither (Fig. 1d–f). However, a previous study showed that 4-1BB co-stimulation retains its ability to enhance central memory accumulation and persistence when CAR-T cells are activated by antigen-coated beads.^1^ Unlike the beads, tumor cells can express PD-L1 in response to interferon-γ (IFN-γ). Indeed, PD-L1 was highly induced in tumor cells in the co-culture system (Supplementary Fig. 3c). Meanwhile, PD-1 was upregulated in CAR-T cells, but its levels were not significantly different between CD8^+^ 28ζ and BBζ cells (Fig. 1g). It has been reported that co-stimulations have little impact on PD-1 expression in CD8^+^ CAR-T cells,^4^ and that PD-1 can induce terminal differentiation of T cells.^3^ Thus, we wondered whether PD-1 was associated with the compromised central memory differentiation and persistence of BBζ cells.

To address this question, PD-1 was blocked by specific antibodies. The cytotoxicity of CAR-T cells with either co-stimulations was similarly improved by PD-1 blockade (*P* < 0.05; Supplementary Fig. 4a, b). But the central memory differentiation was more significantly enhanced in BBζ cells than 28ζ counterparts by PD-1 blockade (*P* < 0.01; Fig. 1h). Correspondingly, the long-term accumulation and tumoricidal effects were better improved in BBζ cells than in 28ζ cells after PD-1 blockade (*P* < 0.05; Fig. 1i, j). To further test the impact of PD-1 signaling induced by PD-L1 on memory differentiation, CAR-T cells were co-cultured with prefixed tumor cells, which had high or negligible PD-L1 expression (Supplementary Fig. 5a). As shown in Supplementary Fig. 5b, c, BBζ cells demonstrated enhanced central memory differentiation and long-term accumulation in extended co-cultures with tumor cells having very low PD-L1 expression (*P* < 0.05). However, the difference in differentiation and persistence between 28ζ and BBζ cells was negligible when PD-L1 was present (Supplementary Fig. 5b, c). These results suggest that PD-1, upon PD-L1 engagement, limits the central memory differentiation and diminishes the advantageous persistence of BBζ cells. Oxidative phosphorylation (OXPHOS) course is a determinant for the central memory differentiation of CAR-T cells.^1^ It was noticed that the privileged OXPHOS potentials of BBζ cells were abrogated when PD-1 was activated (Supplementary Fig. 6a). Further analyses showed that the central memory enrichment and persistence of CAR-T cells were significantly decreased when OXPHOS was inhibited, regardless of co-signals and PD-1 blockade (*P* < 0.05; Supplementary Fig. 6b, c), indicating PD-1 directly suppresses the central memory differentiation by downregulating OXPHOS of CAR-T cells. These findings further suggest that the loss of enhanced central memory differentiation and persistence in 4-1BB co-stimulated CAR-T cells is not due to continuous activation, but instead is the result of PD-1/PD-L1 axis-mediated suppression. Consistent with our findings, PD-1 or PD-L1 blockade was found to efficiently induce augmentation of early lineage and expansion of CD8^+^ T cells during viral infection in mice.^3^

We next performed an in vivo analysis of CAR-T cells. Within xenografted tumors, CAR-T cells were found to be efficiently activated (Supplementary Fig. 7a), while PD-1 and PD-L1 levels were induced in CAR-T cells and tumor cells, respectively (Supplementary Figs. 7b and 8). PD-1 expression levels were similar between 28ζ and BBζ cells (Supplementary Fig. 7b). As expected, memory differentiation, accumulation, and anti-tumor functions of CAR-T cells with CD28 or 4-1BB co-stimulation were indistinguishable when PD-1 was activated in mice treated with a non-specific antibody (Fig. 1k–n). After PD-1 blockade with a neutralizing antibody (Supplementary Fig. 7b), the central memory polarization and accumulation of CAR-T cells were improved compared with those observed in mice treated with the non-specific IgG antibody (*P* < 0.05; Fig. 1k, l). Moreover, in agreement with the in vitro findings, the in vivo central memory polarization and persistence of BBζ cells were better enhanced than those of the 28ζ cells after PD-1 blockade (*P* < 0.05; Fig. 1k, l). Consequently, BBζ cells significantly delayed tumor growth and prolonged mouse survival relative to 28ζ cells (*P* < 0.05; Fig. 1m, n), although cytotoxic activity was similar between both CAR-T cells after PD-1 blockade (Supplementary Fig. 7a).

Preclinical and clinical studies have noted that 4-1BB co-stimulation does not necessarily extend the persistence of CAR-T cells more than that induced by CD28 in vivo,^2,4^ even though it promotes central memory differentiation and extension of the engineered cells in vitro.^1^ Studies on mesothelin-specific CAR-T cells demonstrated that CAR-T cells with 4-1BB co-stimulation exhibit prolonged persistence compared with that of CD28-co-stimulated CAR-T cells under in vitro conditions but not in the immunosuppressive tumor microenvironment.^1,4^ Similarly, studies on CD19-directed CAR-T cell treatment of Nalm-6 xenograft tumors showed that CAR-T cells with 4-1BB co-stimulation exhibit better in vivo persistence than those with CD28 co-stimulation in the less immune-suppressive peripheral blood but not in the more suppressive bone marrow.^5^ These studies indicate that immunosuppression has a critical role in decreasing the difference in memory differentiation and persistence of CAR-T cells in response to different co-stimulations. Our study demonstrates how PD-1-induced suppression on central memory differentiation compromises the persistence and anti-tumor effects of CAR-T cells. PD-1 blockade can recover the advantage observed in 4-1BB-incorporated CAR-T cells in terms of central memory maintenance, in vivo persistence, and long-term anti-tumor effects. Several approaches for decreasing PD-1 levels have already been tested in CAR-T cell therapy.^4^ Thus, a combination of PD-1 blockade and CAR-T cells with 4-1BB co-stimulation may potentially lead to better therapeutic responses.

## Supplementary information

Supplementary Material

## Data Availability

All supporting data are included in the manuscript and supplemental files. Additional data are available upon reasonable request to the corresponding author.
