# Supplementary material for: PD-1 abrogates the prolonged persistence of CD8+ CAR-T cells with 4-1BB co-stimulation
Source: Signal Transduct Target Ther. 2020 Aug 25;5:164. doi: 10.1038/s41392-020-00277-6 (PMC7447768; doi:10.1038/s41392-020-00277-6)
Supplement: Supplementary file 1 — Supplementary Material [file 41392_2020_277_MOESM1_ESM.docx]

Supplementary Materials for

PD-1 abrogates the prolonged persistence of CD8+ CAR-T cells with 4-1BB co-stimulation

Feng Li^1,2,4,5,a,^*, Zhen Zhang^1,2,4,5,a,^, Yujing Xuan^1,2,4,a,^, Daiqun Zhang^1,2,3,4^, Jinyan Liu^1,2,4^, Aitian Li^1,2,4^, Shumin Wang^1,2,4^, Ting Li^1,2,4^, Xiaojuan Shi^1,2,4^, Yi Zhang^1,2,3,4,5,^*

Correspondence to: [yizhang@zzu.edu.cn](mailto:yizhang@zzu.edu.cn); [lifeng01@msn.com](mailto:lifeng01@msn.com)

**This PDF file includes:**

Materials and Methods

Figures. S1 to S8

Materials and Methods

**Cell lines, media, and reagents**

Human 293T, A549, H322, HeLa, and Jurkat cell lines were obtained from the Cell Bank of the Chinese Academy of Sciences (Shanghai, China). Cell lines were maintained in DMEM media (Gibco; Thermo Fisher Scientific, Waltham. MA) supplemented with 10% fetal bovine serum (FBS; Hyclone, Logan, TX), 1% antibiotics (penicillin and streptomycin; Gibco), and anti-mycoplasma agents (InvivoGen, San Diego, CA) at 37 °C and 5% CO_2_. Non-specific IgG antibody, anti-PD-1 neutralizing antibody, and rotenone were obtained from MedChemExpress (Monmouth Junction, NJ). All other chemicals were obtained from Sigma-Aldrich (St. Louis, MO) unless otherwise indicated.

**Lentivirus production and T cell infection**

The sequence coding for the mesothelin-binding region was derived from a human-sourced antibody ^1^. Together with other components ^2^, the coding sequences of CAR were synthesized. Then, DNA fragments coding 28ζ or BBζ were inserted into the pCDH lentiviral vector (Systembio, Mountain View, CA) and lentivirus production was carried out as previously reported ^3^. Briefly, when 293T cell confluency reached 80%, we transfected CAR-coding vectors with helper vectors (psPAX2 and pMD2.G) using CaPO_4_ precipitation. After 6 hours, the supernatants were replaced with fresh media. After another 48 hours, the supernatants containing mature lentivirus were collected and stored at 4 °C. CD8^+^ T cells were purified from peripheral blood mononuclear cells (PBMCs) of healthy donors using positive selection kits (Miltenyi Biotech, Bergisch Gladbach, Germany), after which the T cells were stimulated with anti-CD3/CD28 Dynabeads (Invitrogen, Carlsbad, CA) in the presence of 200 IU/mL interleukin-2 (IL-2) for 2 days. The spinfection protocol was adopted to increase transgene efficacy. After incubating with lentivirus-containing media and polybrene (6 μg/mL), activated T cells were centrifuged at 1000 × g for 2 hours at 32 °C. Following infection, T cells were expanded in RPMI1640 media supplemented with 5% FBS and IL-2 (200 IU/mL), and counted every 2 days. CAR-T cells were used for *in vitro* and *in vivo* experiments 14 days post infection.

**FACS assay**

Fluorescence-activated cell sorting (FACS) was carried out as previously described ^3^. Briefly, single cell suspensions were prepared, collected, and washed with ice-cold PBS containing 2% FBS. Then, fluorochrome-conjugated antibodies against human CD45, CD8, CD45RA, CD62L, CD69, PD-1, PD-L1, or EpCAM (BioLegend, San Diego, CA) were added to the samples and incubated for 30 minutes on ice. Fixable viability dye eFluor™ 660 (Invitrogen) was added to exclude dead cells. Cells were then washed three times with PBS containing 2% FBS and sorted using a BD Canto II cytometer (Becton Dickinson, Franklin Lakes, NJ). For each analysis, at least 10000 target cells were collected and further analyzed with FlowJo software (Tree Star Inc., Ashland, OR).

**Cytolytic function analysis of CAR-T cells**

The cytolytic function of CAR-T cells was determined via a bioluminescent imaging (BLI) assay on an IVIS Spectrum Imaging System (PerkinElmer, Waltham, MA). Target cells expressing firefly luciferase were incubated with CAR-T cells at various ratios of effector to target (E:T) for 24 hours, after which the viability of target cells was monitored according to BLI intensity.

**Cytokine production of CAR-T cells**

*ELISA detection*

CAR-T cells were washed with RPMI1640 media and then mixed with target cells at an E:T ratio of 1:1 for 24 hours without exogenous cytokines. The supernatants were then collected and tested for interferon-γ (IFN-γ) and IL-2 secretion using cytokine-specific detection kits (BioLegend) according to manufacturer’s instructions.

*Intracellular staining*

Detecting the intracellular production of IFN-γ and IL-2 was performed according to manufacturer’s instructions. Briefly, CAR-T cells were collected, washed in ice-cold PBS with 2% FBS, fixed, and permeabilized. Then, cells were incubated with fluorochrome-conjugated antibodies (BioLegend) for 30 minutes on ice. After washing in ice-cold PBS with 2% FBS, the cells were analyzed using a flow cytometer.

**Extended co-culture assay**

Luciferase-expressing tumor cells were plated in advance, after which purified CAR-T cells were added at an E:T ratio of 1:10 into tumor cells without additional cytokines. On day 3 and 7, CAR-T cells in the co-culture systems were collected after gently pipetting up and down, counted after exclusion of dead cells by Trypan Blue staining, and then stained with Fixable Viability Dye eFluor™ 660 and fluorochrome-conjugated antibodies as described above. The phenotypes of GFP^+^ cells were determined with a flow cytometer. Meanwhile, the viability of tumor cells was monitored via the BLI assay. In experiments exploring the effects of PD-L1 on T cell differentiation, tumor cells were fixed in paraformaldehyde first and then mixed with CAR-T cells at an E:T ratio of 1:10.

**Metabolic analysis**

The metabolic alterations of glucose degradation in T cells were tested as reported previously ^4^. Briefly, CAR-T cells activated for 3 days were seeded into microplates coated with Cell-Tak (Corning) for adhesion. Then the oxygen consumption rate (OCR) (pmoles/min) was tested. T cells were suspended in XF assay media containing 5.5 mM glucose and 1 mM pyruvate, and oligomycin (0.75 μM), carbonyl cyanide-4-(trifluoromethoxy)phenylhydrazone (FCCP; 1 μM) and rotenone and antimycin (both 1 μM) were injected sequentially. All chemicals were purchased from Sigma-Aldrich. Calculations for each parameters were per manufacturer’s instructions (Seahorse Bioscience).

**Mouse xenograft model**

Female SCID-Beige mice aged 6-8 weeks were obtained from Beijing Vital River Laboratory Animal Technology Co., Ltd. (China). Mice were subcutaneously inoculated with 2 × 10^6^ HeLa cells that were suspended in PBS. After 10 days, 5 × 10^6^ CD8^+^ untransduced (UTD) or CAR-T cells were injected through the tail vein. Meanwhile, IgG or anti-PD-1 neutralizing antibodies were intraperitoneally injected at 200 µg per mouse, six times every 3 days. After T cell injection, tumor volumes were calculated as previously described ^5^. Once the volumes reached 1500 mm^3^, the mice were sacrificed. All procedures were carried out according to the protocol approved by the Institute Ethics Committee of the First Affiliated Hospital of Zhengzhou University (Zhengzhou, China).

**Data analysis**

Statistical analyses were performed using GraphPad Prism version 7 (GraphPad Software Inc., La Jolla, CA). Data are presented as the mean ± SD of at least three independent experiments. For comparing differences between two groups, a Student’s *t*-test was performed and for comparing three or more groups, we employed one- or two-way ANOVA. *P* values < 0.05 were considered statistically significant.

**References**

1 Ho, M., Feng, M., Fisher, R. J., Rader, C. & Pastan, I. A novel high-affinity human monoclonal antibody to mesothelin. *Int. J. Cancer* **128**, 2020-2030 (2011).

2 Carpenito, C. *et al.* Control of large, established tumor xenografts with genetically retargeted human T cells containing CD28 and CD137 domains. *Proc. Natl. Acad. Sci. U. S. A.* **106**, 3360-3365 (2009).

3 Shi, X. *et al.* Targeting glycosylation of PD-1 to enhance CAR-T cell cytotoxicity. *J. Hematol. Oncol.* **12**, 127 (2019).

4 Zhang, T, *et al*. miR-143 regulates Mmemory T cell differentiation by reprogramming T cell metabolism. *J. Immunol.* **201**, 2165-2175 (2018).

5 Li, F. *et al.* RhoA modulates functional and physical interaction between ROCK1 and Erk1/2 in selenite-induced apoptosis of leukaemia cells. *Cell Death Dis.* **4**, e708 (2013).


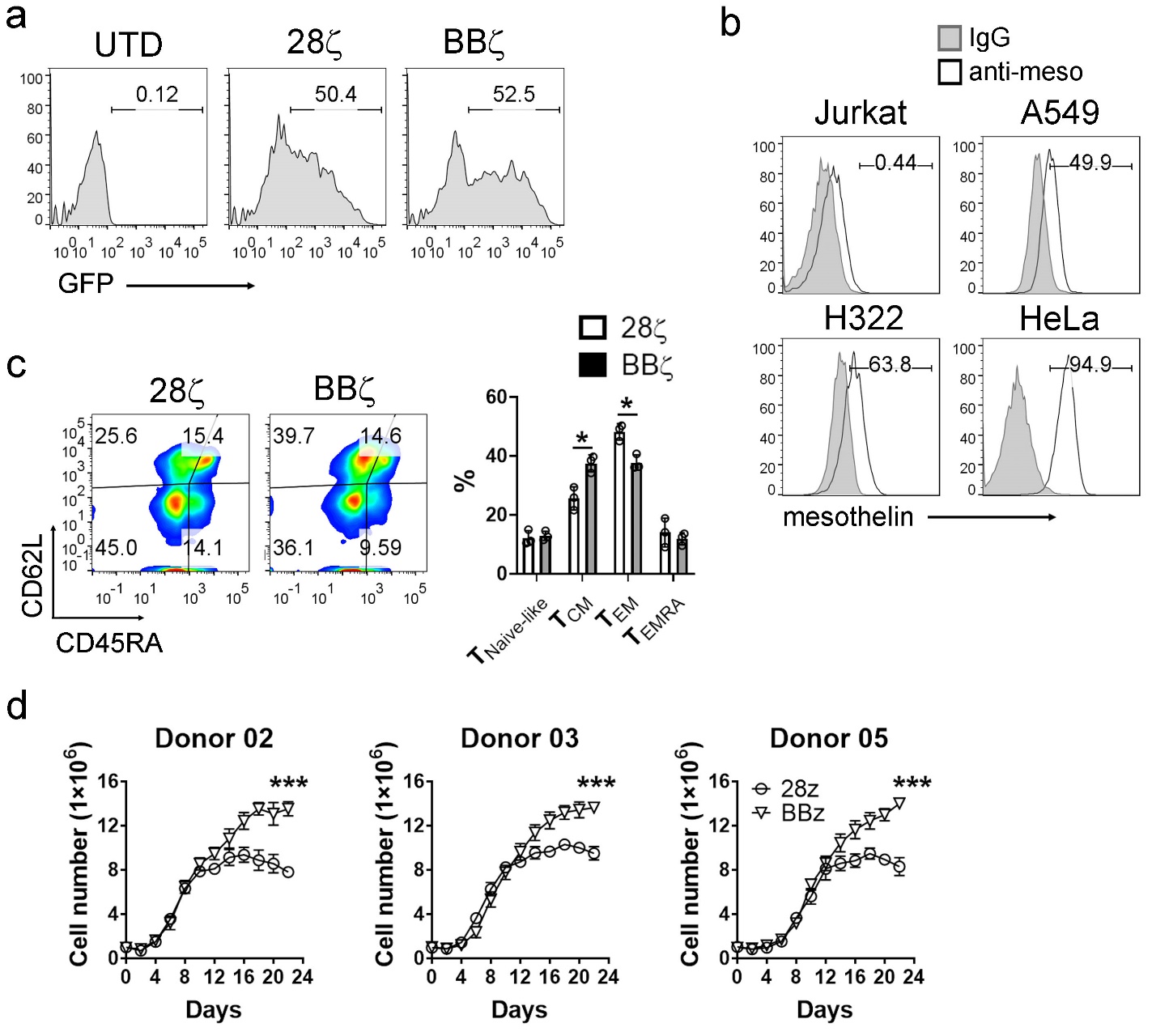


Figure. S1.

Proliferation and memory differentiation of CD8^+^ CAR-T cells during *in vitro* expansion. a. CD8^+^ T cells were transduced with lentivirus coding 28ζ or BBζ. Five days later, the transduction efficacies were determined using FACS. b. Mesothelin expression on various tumor cell lines. c. Differentiation status of CAR-T cells on day 14 post transduction. Among 28ζ and BBζ cells, the ratios of naive T (T_Naive-like_; CD45RA^+^CD62L^+^), T_CM_ (CD45RA^-^CD62L^+^), T_EM_ (CD45RA^-^CD62L^-^), and most differentiated T (T_EMRA_; CD45RA^+^CD62L^-^) cells were statistically analyzed. d. Proliferation of CD8^+^ 28ζ and BBζ T cells from different donors on the indicated days post transduction. At the indicated time points, CAR-T cells were collected and stained with Trypan Blue dye, after which live cells were counted. Data presented as the means ± SD are representative of three independent tests on samples collected from seven healthy donors. Statistical analysis was performed using *t*-tests. *P* values < 0.05 are considered statistically significant. **P* < 0.05; ****P* < 0.005.


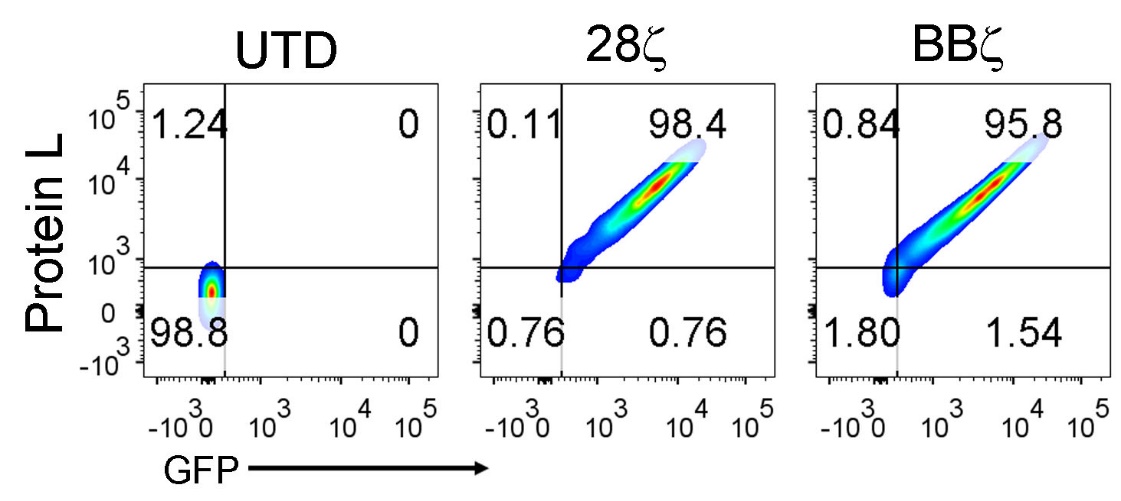


Figure. S2.

Purification of CD8^+^ CAR-T cells. Fourteen days after transduction, CAR-T cells were purified using a FACS sorter. The surface expression of CAR was determined in sorted cells with Protein L staining. Purified CAR-T cells were used in subsequent experiments.


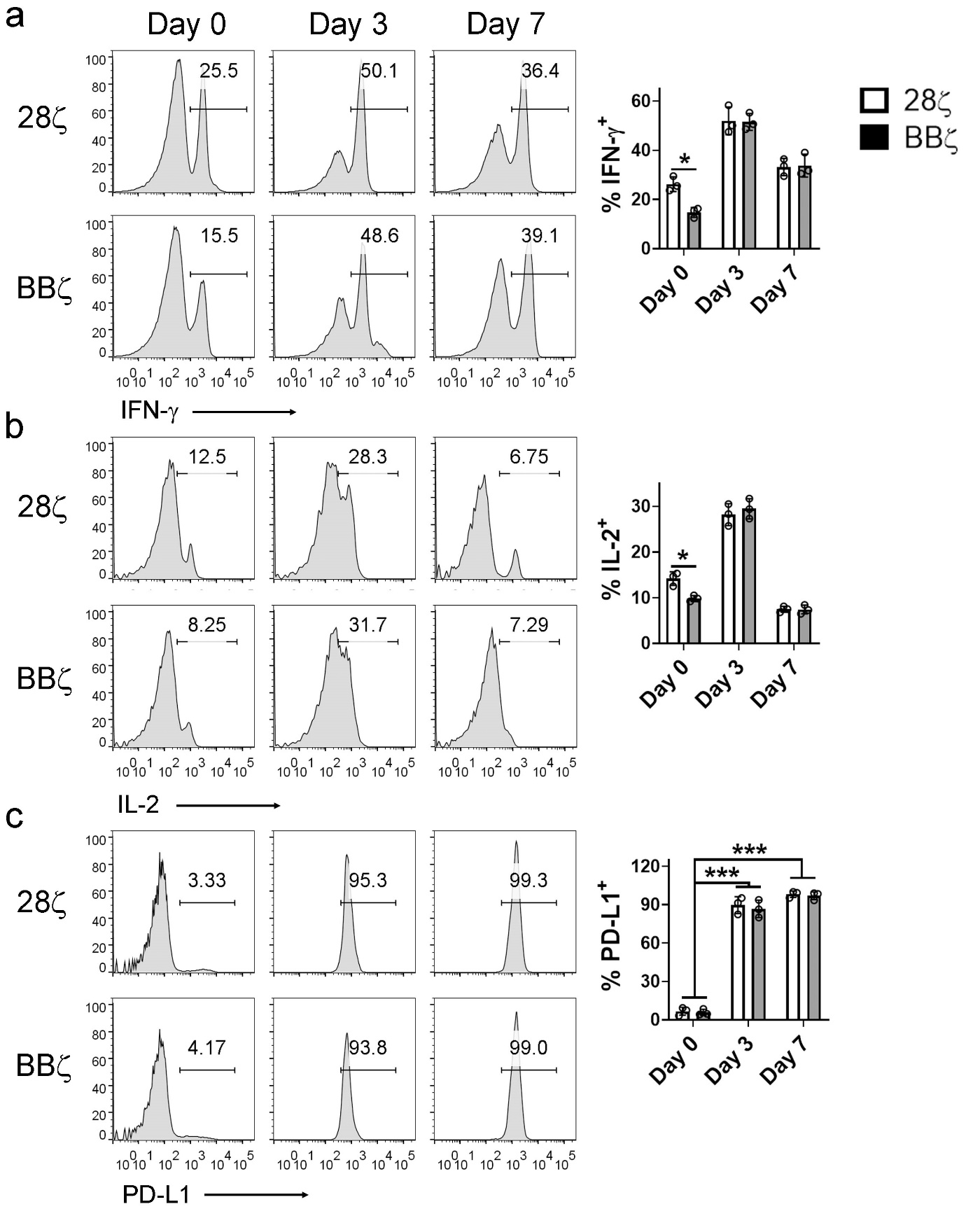


Figure. S3.

Antigen-dependent activation of CAR-T cells. Luciferase-expressing HeLa cells were pre-plated overnight, then purified CD8^+^ CAR-T cells were added at E:T = 1:10 without exogenous cytokines. **a** and b. On day 7, CAR-T cells were collected and intracellular IFN-γ (**a**) and IL-2 (**b**) levels were determined using FACS. **c**. PD-L1 expression during co-culture. On day 0, 3, and 7 post CAR-T cell addition, tumor cells were collected and stained with specific fluorochrome-conjugated antibodies. Then expression levels of PD-L1 were determined using FACS. Data are representative of three independent tests on samples collected from seven healthy donors. Statistical analysis was performed using ANOVA. *P* values < 0.05 are considered statistically significant. **P* < 0.05; ****P* < 0.005.


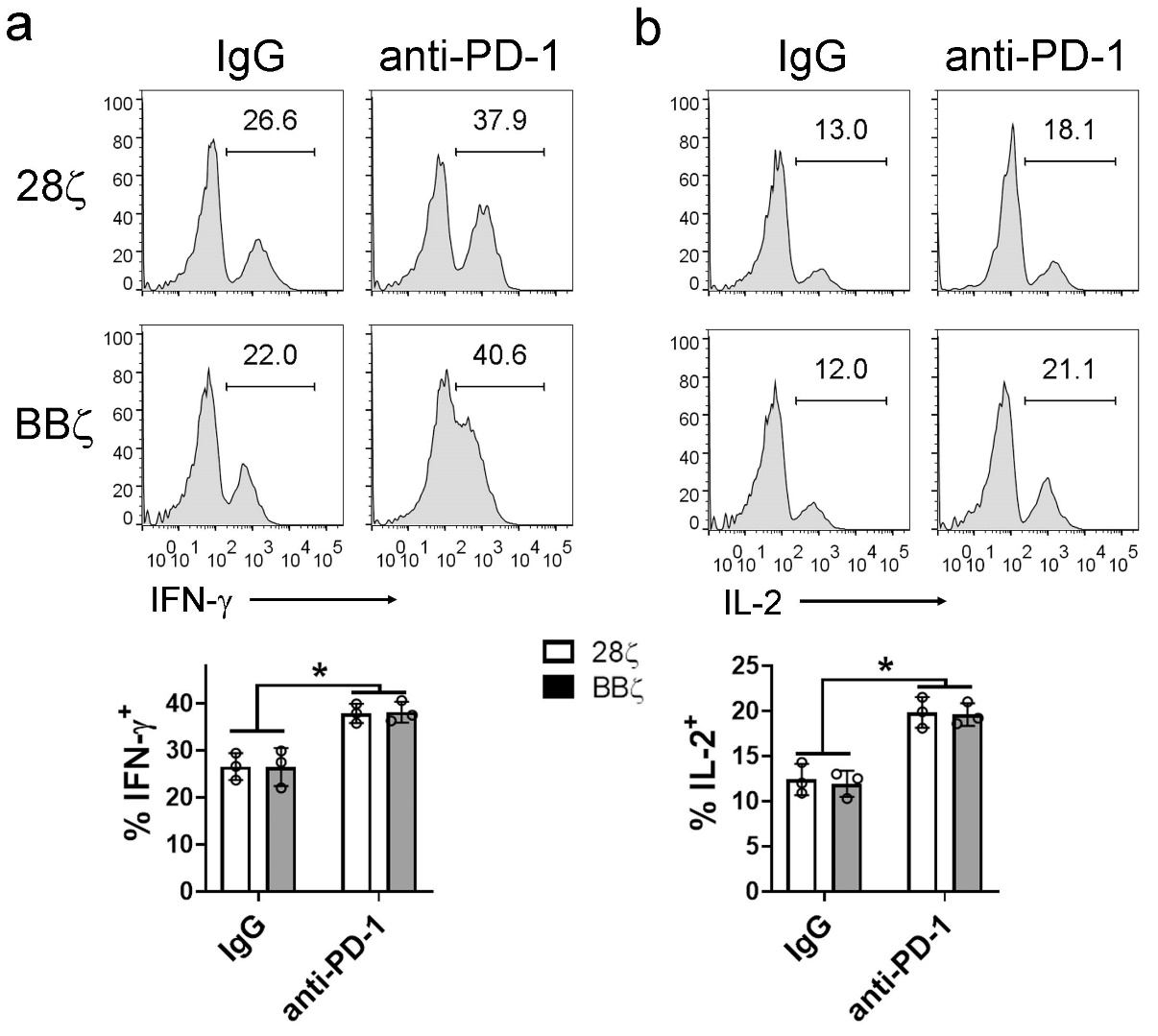


Figure. S4.

PD-1 blockade recovers the persistence of CD8^+^ CAR-T cells. Following luciferase-expressing HeLa cells were adhered overnight, purified CAR-T cells were added at E:T = 1:10 without exogenous cytokines. IgG and anti-PD-1 antibody were repeatedly added on day 0, 3, and 6 at a concentration of 20 μg/mL. **a** and **b**. On day 7, CAR-T cells were collected and their intracellular productions of IFN-γ (**a**) and IL-2 (**b**) were determined via FACS. Data are representative of three independent experiments on CAR-T cells collected from 3 healthy donors. Statistical analysis was performed using ANOVA. *P* values < 0.05 are considered statistically significant. **P* < 0.05.


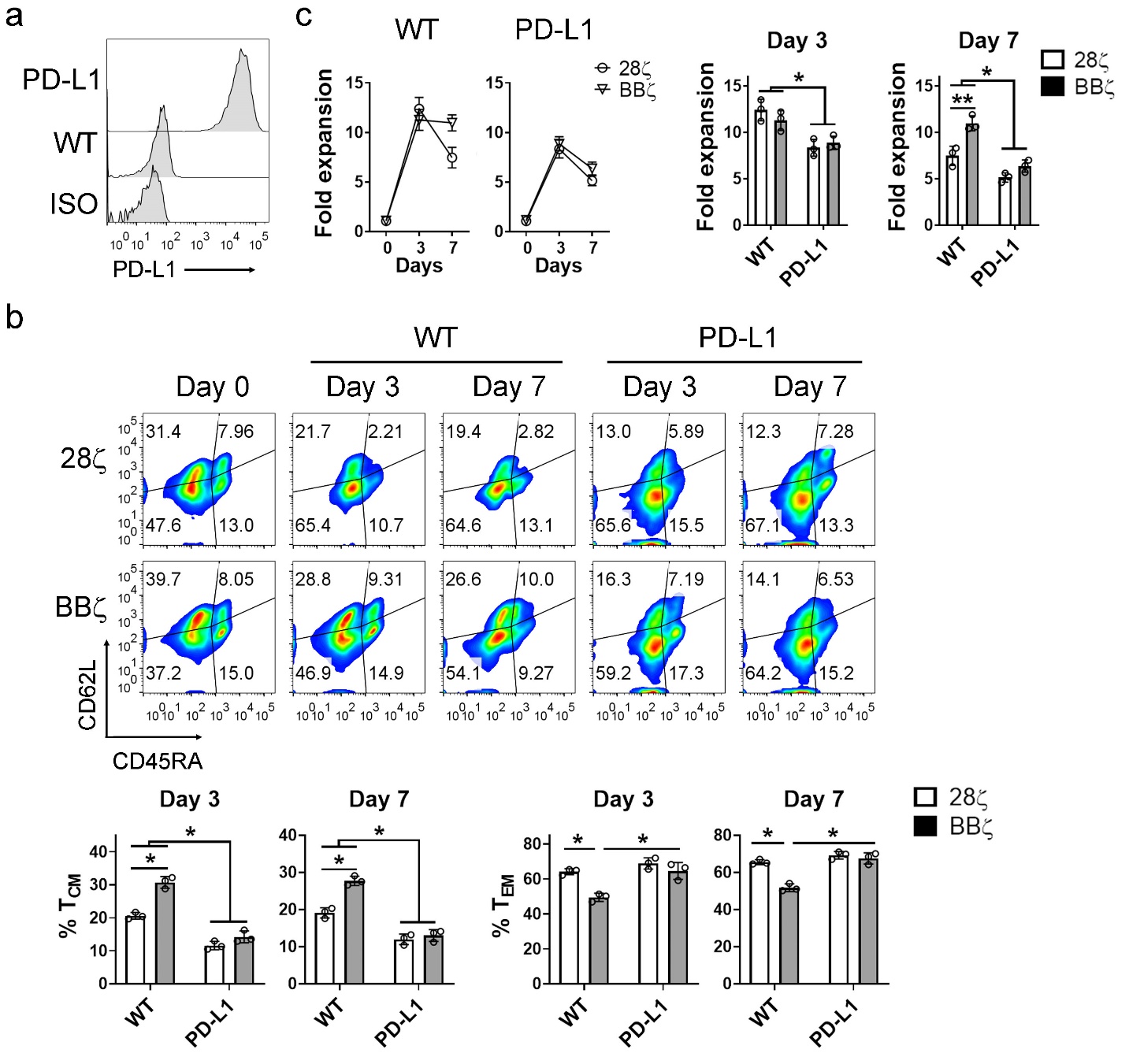


Figure. S5.

PD-L1 affects T cell differentiation. **a**. Surface expression of PD-L1 on HeLa cells overexpressing vector (WT) or gene of interest (PD-L1). **b**. Purified CD8^+^ CAR-T cells were mixed with pre-fixed HeLa cells at E:T = 1:10, and freshly fixed HeLa cells were re-supplemented every 3 days. CAR-T cell differentiation was analyzed after engagement with WT or PD-L1 target cells, and the T_CM_ and T_EM_ subsets were statistically analyzed. **c**. The numbers of CAR-T cells were counted and the expanding indices were statistically compared on the indicated days. Data are representative of three independent experiments on CAR-T cells collected from 3 healthy donors. Statistical analysis was performed using ANOVA. *P* values < 0.05 are considered statistically significant. **P* < 0.05.


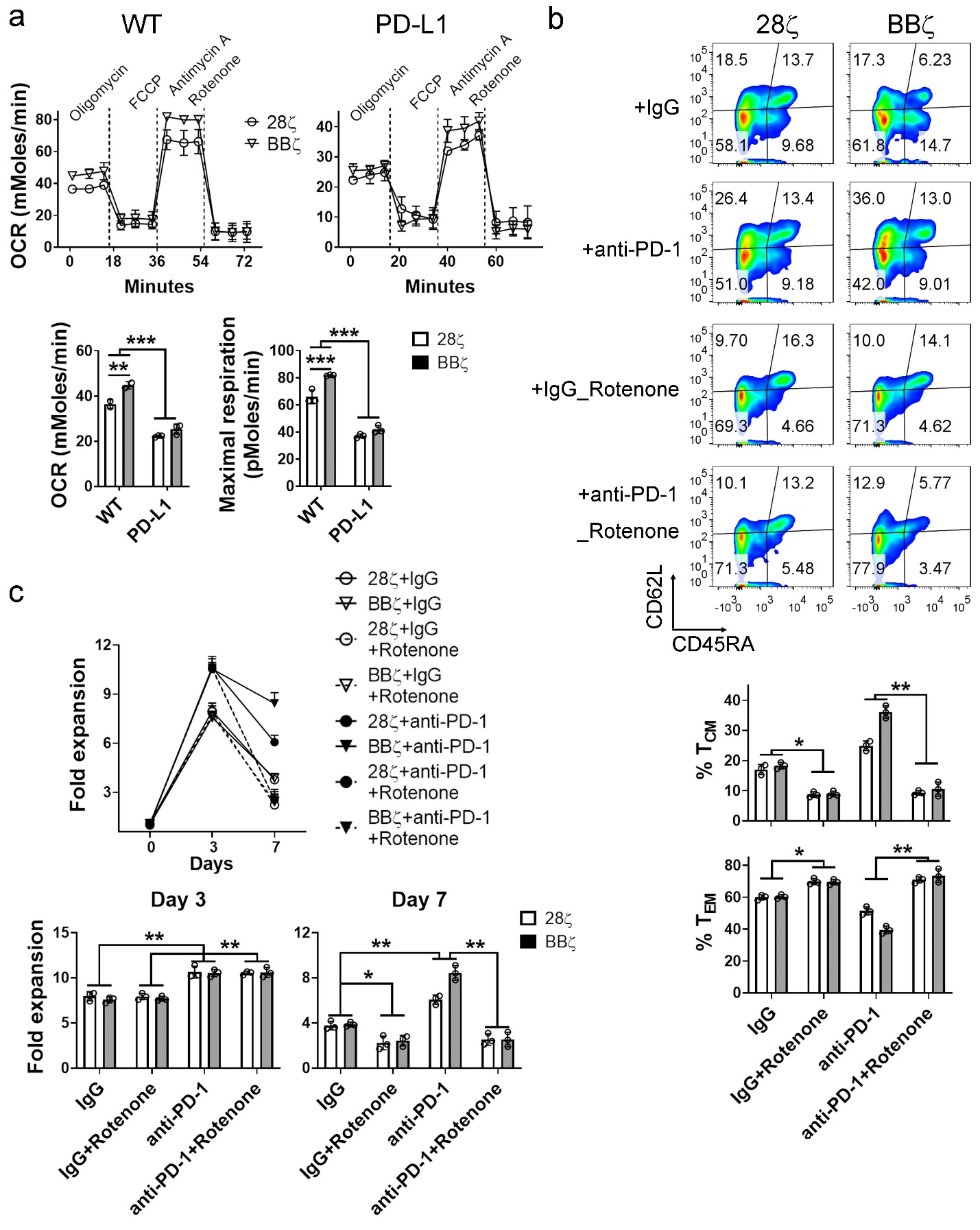


Figure. S6.

OXPHOS is critical for central memory maintenance of CAR-T cells. **a**. Purified CAR-T cells were co-incubated with pre-fixed HeLa cells with different PD-L1 expressions without exogenous cytokines. On day 3 post incubation, CAR-T cells were collected and probed for the oxygen consumption rate (OCR), which indicated the potentials of OXPHOS. **b** and **c**. After HeLa cells were adherent overnight, CAR-T cells and the indicated reagents were added on day 0. The IgG or PD-1-specific antibody (20 μg/mL) and Rotenone (5 nM) were re-supplied on day 3 and day 6. On day 7 post incubation, the memory differentiation of CAR-T cells from different groups was checked (**b**). The proliferations of CAR-T cells were analyzed at indicated times (**c**). Data shown are representative of independent assays with CAR-T cells from 3 health donors. ANOVA tests were performed and *P* < 0.05 indicated statistical significance. **P* < 0.05; ***P* < 0.01; ****P* < 0.005.


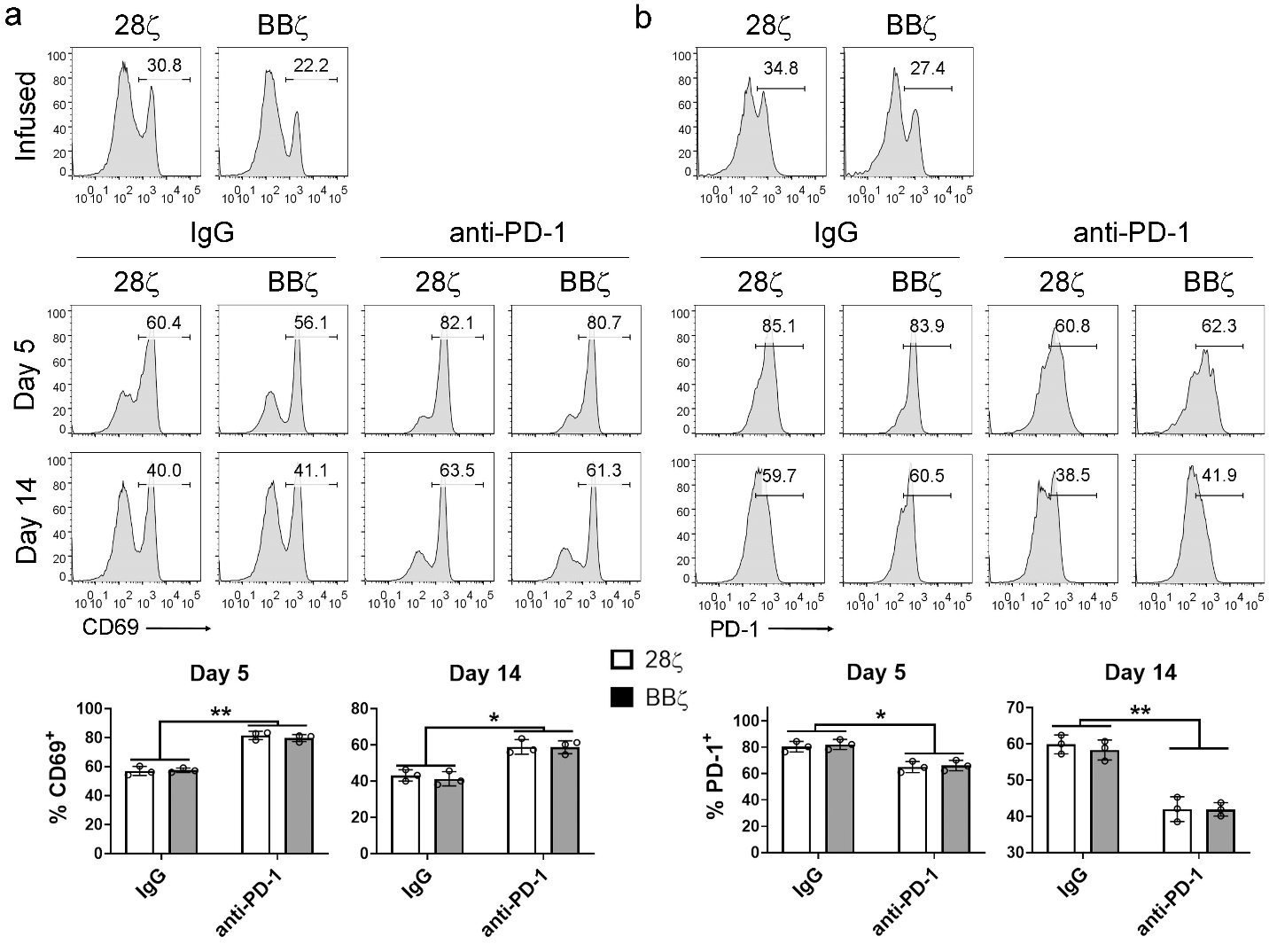


Figure. S7.

PD-1 expression in CD8^+^ CAR-T cells *in vivo*. Ten days post HeLa cells were subcutaneously inoculated into SCID-Beige mice, CD8^+^ CAR-T cells were injected through the tail vein and the indicated antibodies were injected intraperitoneally four times every 3 days (n = 3 per group). On day 5 and 14 post CAR-T cell infusion, tumor tissues were isolated and dispersed. After excluding dead cells using Fixable Viability Dye eFluor™ 660, the activating levels (**a**) and PD-1 expression (**b**) of CAR-T cells were determined between mice that received non-specific IgG or anti-PD-1 neutralizing antibody. CAR-T cells for infusion were used as control. Statistical analysis was performed using ANOVA. *P* < 0.05 represents statistical significance. **P* < 0.05; ***P* < 0.01.


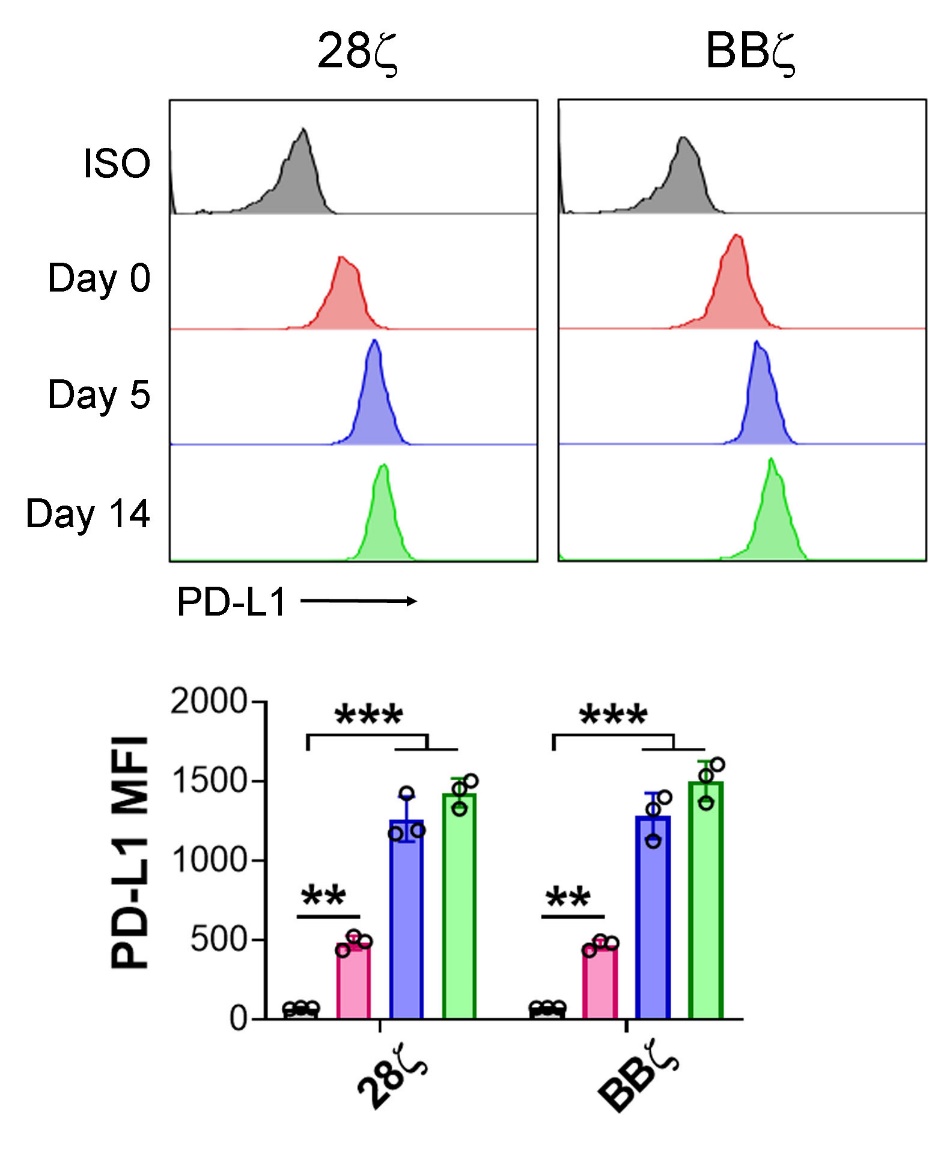


Figure. S8.

PD-L1 expression in xenograft tumor tissues. Tumor cells were isolated at the indicated time points and PD-L1 expression was detected using FACS. Statistical analysis was performed using ANOVA. *P* < 0.05 represents statistical significance. ***P* < 0.01; ****P* < 0.005.
